# Gangrenous appendicitis within a long-standing umbilical hernia: a case report

**DOI:** 10.1093/jscr/rjag166

**Published:** 2026-03-17

**Authors:** Feruzjon G Bobosharipov, Yulduz I Nadirova

**Affiliations:** Department of Surgical Diseases in Family Medicine, Tashkent State Medical University, Tashkent, Uzbekistan; Department of Faculty and Hospital Therapy No2, Nephrology and Hemodialysis, Tashkent State Medical University, Tashkent, Uzbekistan

**Keywords:** umbilical hernia, appendicitis, incarcerated hernia, gangrenous appendix

## Abstract

Appendicitis occurring within an umbilical or paraumbilical hernia is an extremely rare clinical entity and poses a significant diagnostic challenge. Because umbilical hernia sacs usually contain omentum or bowel, the presence of the appendix may obscure classical features of appendicitis and mimic a strangulated hernia. We report the case of a 63-year-old woman with a 29-year history of umbilical hernia who presented with acute pain, erythema, and an irreducible umbilical mass. She underwent emergency surgical exploration, which revealed gangrenous omentum and a gangrenous appendix measuring 10 × 2 cm within the hernia sac. Appendectomy, omentectomy, and primary layered hernia repair were performed. The postoperative course was uneventful. This case highlights that appendicitis within an umbilical hernia, although rare, should be considered in patients presenting with an acutely painful irreducible umbilical hernia, as prompt surgical intervention leads to excellent outcomes.

## Introduction

Umbilical and paraumbilical hernias typically contain omentum and small bowel, whereas involvement of the appendix is extremely uncommon [1, 2]. The presence of the appendix has been more frequently described in femoral (De Garengeot) and inguinal hernias (Amyand hernia), while its occurrence in umbilical hernias is rare, with only isolated reports worldwide [3]. The rarity is attributed to the usual anatomic location of the appendix and its limited mobility. When inflammation occurs within a hernia sac, it can mimic incarcerated or strangulated hernia clinically, delaying the diagnosis of appendicitis [4, 8]. We describe a rare case of gangrenous appendicitis within a long-standing umbilical hernia in an adult female, and we contextualize the case using available literature.

## Case report

**Figure 1:**
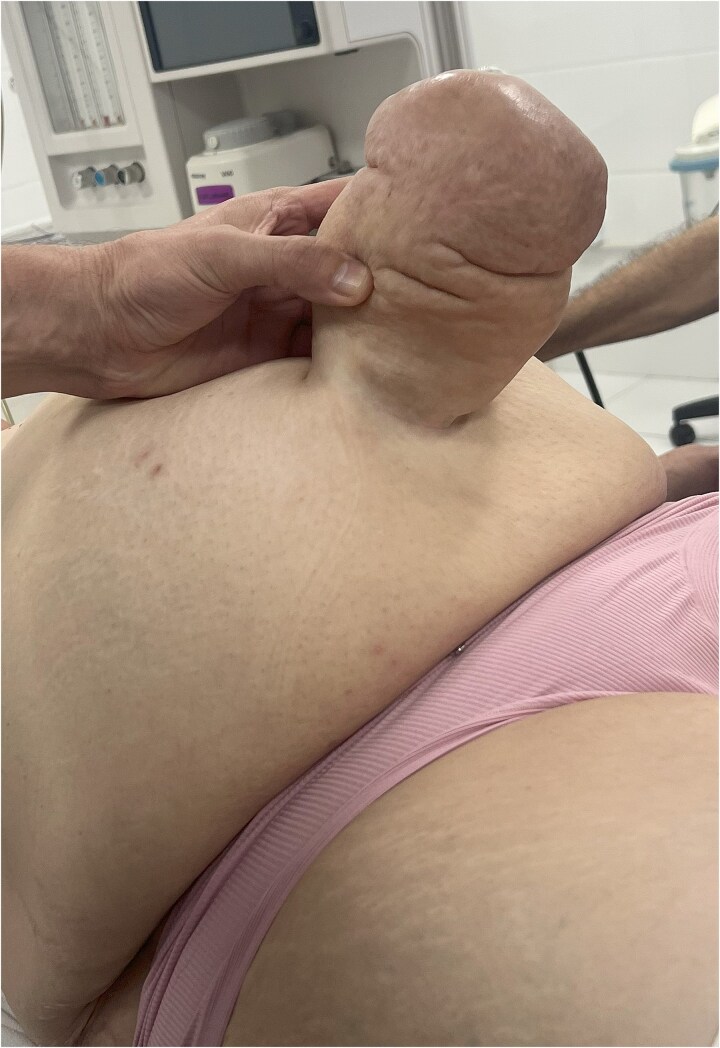
Preoperative appearance of the patient’s umbilical hernia.

We are presenting a case of a 63-year-old woman presented to the emergency department with a 5–6-hour history of acute umbilical pain accompanied by nausea and progressive localized tenderness. She reported a 29-year history of an umbilical hernia that had previously been fully reducible but became irreducible during the current episode. Her past medical history was notable only for type 2 diabetes mellitus. She had no history of prior abdominal surgery and denied smoking or any other harmful habits. Her body mass index was calculated as 38.0 kg/m^2^ (95 kg, 158 cm), consistent with obesity. On clinical examination, vital signs revealed blood pressure of 90/60 mmHg, pulse of 96 beats/min, temperature of 36.8°C, and respiratory rate of 18/min. Abdominal evaluation demonstrated a 15 × 15 cm irreducible and tender umbilical hernia with erythematous overlying skin, though without signs of generalized peritonitis. Bowel sounds were preserved (Fig. 1). Laboratory investigations showed leukocytosis (WBC 14 × 10^9^/L) with normal haemoglobin (131 g/L). Ultrasound and plain abdominal radiography demonstrated an umbilical hernia containing omentum and small bowel loops, without evidence of pneumoperitoneum, consistent with hernia incarceration.

**Figure 2:**
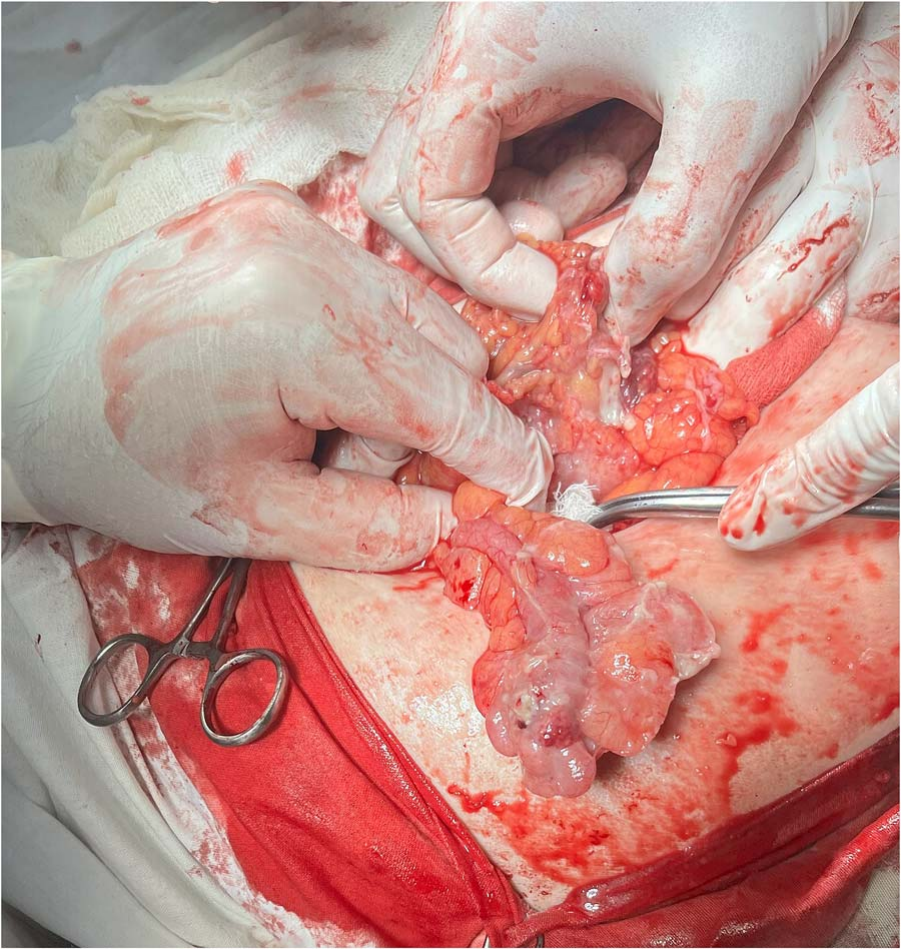
Intraoperative view showing the inflamed appendix freed from the hernial sac.

The patient underwent emergency herniolaparotomy. Intraoperative findings included ischemic and inflamed omentum and a markedly enlarged, gangrenous appendix measuring 10 cm in length and 2 cm in diameter located within the hernia sac (Fig. 2). There was no bowel perforation. An incidental uterine leiomyoma was also noted. Surgical management consisted of omentectomy, appendectomy, and primary hernia repair using a layer-by-layer Mayo technique. An intra-abdominal drain was placed. Mesh was deliberately avoided because of contamination risk, consistent with recommendations for infected or potentially infected operative fields. The postoperative course was uneventful. The patient received antibiotic therapy, underwent routine wound care, and experienced no postoperative complications. Histopathological examination confirmed acute gangrenous appendicitis.

## Discussion

Appendicitis occurring within an umbilical or paraumbilical hernia sac represents a rare and clinically important condition. Only a small number of such cases have been documented, and a systematic review identified merely six cases reported over a 12-year period, reflecting the exceptional nature of this presentation [3, 4]. Its rarity contributes to diagnostic difficulty, yet early recognition is essential because delayed treatment can lead to ischemia, perforation, or sepsis [4].

The underlying pathophysiology remains incompletely defined, but several plausible mechanisms have been proposed. Increased mobility of the appendix due to anatomical variations may facilitate its migration into the hernia sac [3]. Once within the sac, extrinsic compression at the narrow neck may impair venous drainage, resulting in congestion, ischemia, and subsequent inflammatory changes [1, 3]. Incarceration of the hernia itself may further exacerbate vascular compromise, producing secondary appendicitis [2, 8]. These mechanisms are supported by multiple reports describing both ventral hernias containing the appendix [1] and cases of paraumbilical hernia-associated appendicitis [3].

The diagnostic process is often challenging, as the clinical presentation closely resembles that of an incarcerated or strangulated hernia. Patients typically exhibit acute localized pain, an irreducible mass, and cutaneous erythema, while systemic signs may be absent early in the disease course. Imaging modalities such as computed tomography can provide valuable diagnostic clues, as reported in previous cases [3, 6, 7]; however, due to subtle or nonspecific radiologic changes, definitive diagnosis is frequently established only at the time of surgery.

Surgical management remains the mainstay of treatment. Standard therapy includes appendectomy combined with hernia repair, with the choice between mesh reinforcement or primary tissue repair dictated by the degree of operative field contamination [5]. Most authors advocate avoiding mesh placement in the presence of gangrenous appendix, abscess, perforation, or purulent contamination, due to increased risk of postoperative infection and mesh-related complications. This recommendation is consistent with the management applied in the present case [1, 4].

Most published cases and systematic analyses strongly advise against mesh placement in the presence of gangrenous appendix, perforation, abscess formation, or purulent contamination due to the increased risk of postoperative infection and mesh-related complications [1, 3, 5]. This recommendation aligns with the management strategy implemented in the present case.

Multiple authors have documented presentations similar to ours. Zormpa *et al.* reported a perforated appendix within a paraumbilical hernia, characterized by localized erythema and an irreducible mass [4]. Sigley *et al.* described a necrotic appendix incarcerated in an umbilical hernia requiring open appendectomy and hernia repair [5].

Our case differs from previously reported ones due to the presence of a long-standing umbilical hernia of 29 years’ duration and the discovery of a markedly elongated, 10-cm gangrenous appendix. This combination of chronic hernia history and severe appendiceal pathology further underscores the uniqueness of the presentation and the importance of timely surgical assessment in patients with atypical abdominal wall masses.

In conclusion, appendicitis developing inside an umbilical hernia sac is extremely uncommon but remains a condition of notable clinical importance. It should be considered in adults who present with an acutely painful, irreducible umbilical hernia, especially in the context of long-standing defects. Prompt surgical exploration remains the key to accurate diagnosis and is associated with excellent postoperative outcomes.

## Data Availability

All data generated or analyzed during this study are included in this published article.

## References

[1] Pilia T, Murzi V, Locci E et al. Where have I lost my appendix? An unusual finding of acute appendicitis within an irreducible umbilical hernia sac. Case Report Acta Biomed 2024;95:e2024049. 10.23750/abm.v95i3.14598

[2] Agarwal N, Goyal S, Kumar A et al. Appendicitis in paraumbilical hernia mimicking strangulation: a case report and review of the literature. Hernia 2013;17:531–2. 10.1007/s10029-013-1118-3 23708684

[3] Hermus L, Domerchie P, Kloppenberg FWH. Appendicitis in an umbilical hernia: a case report. EC Gastroenterol Digestive Syst 2018;5:920–5.

[4] Zormpa M, Alfa-Wali A, Chung A. Appendicitis within an incarcerated paraumbilical hernia. BMJ Case Rep 2019;12:e228915. 10.1136/bcr-2018-228915 PMC6700550 31401566

[5] Sigley K, Russo T, Welch S. Umbilical hernia containing appendicitis. Cureus 2020;12:e8075. 10.7759/cureus.8075 32542130 PMC7290121

[6] Wani SA, Hassan F, Baba AA et al. Acute perforated appendix mimicking umbilical hernia: a rare case report. J Clin Med Case Rep 2016;3:1–3.

[7] Atabek C, Sürer I, Delibağa H et al. Appendicitis within an umbilical hernia sac: a previously unreported complication in children. Ulus Travma Acil Cerrahi Derg 2008;14:245–6. 18781423

[8] Arnáiz J, Ortiz A, Marco de Lucas E et al. Unusual perforated appendicitis within umbilical hernia: CT findings. Abdom Imaging 2006;31:691–3. 10.1007/s00261-005-8009-8 16465570

